# Deep Learning Predicts the Malignant-Transformation-Free Survival of Oral Potentially Malignant Disorders

**DOI:** 10.3390/cancers13236054

**Published:** 2021-12-01

**Authors:** John Adeoye, Mohamad Koohi-Moghadam, Anthony Wing Ip Lo, Raymond King-Yin Tsang, Velda Ling Yu Chow, Li-Wu Zheng, Siu-Wai Choi, Peter Thomson, Yu-Xiong Su

**Affiliations:** 1 Division of Oral and Maxillofacial Surgery, Faculty of Dentistry, The University of Hong Kong, Hong Kong 999077, China; jaadeoye@hku.hk (J.A.); lwzheng@hku.hk (L.-W.Z.); htswchoi@hku.hk (S.-W.C.); 2 Division of Applied Oral Sciences and Community Dental Care, Faculty of Dentistry, The University of Hong Kong, Hong Kong 999077, China; koohi@hku.hk; 3 Department of Pathology, Queen Mary Hospital, Hong Kong 999077, China; lwi543@ha.org.hk; 4 Division of Otorhinolaryngology, Department of Surgery, Li Ka Shing Faculty of Medicine, The University of Hong Kong, Hong Kong 999077, China; rkytsang@hku.hk; 5 Division of Head and Neck Surgery, Department of Surgery, Li Ka Shing Faculty of Medicine, The University of Hong Kong, Hong Kong 999077, China; cly329@ha.org.hk; 6 College of Medicine and Dentistry, James Cook University, Cairns, QLD 4870, Australia

**Keywords:** artificial intelligence, machine learning, oral leukoplakia, oral lichenoid lesions, oral cancer

## Abstract

**Simple Summary:**

Mouth cancer is the most common malignancy in the head-and-neck region. Usually, these tumors develop from white lesions in the mouth that appear long before cancer diagnosis. However, platforms that can estimate the time-factored risk of cancer occurring from these diseases and guide treatment and monitoring approaches are elusive. To this end, our study presents time-to-event models that are based on machine learning for prediction of the risk of malignancy from oral white lesions following pathological diagnosis as a function of time. These models displayed very satisfactory discrimination and calibration after multiple tests. To facilitate their preliminary use in clinical practice and further validation, we created a website supporting the use of these models to aid decision making.

**Abstract:**

Machine-intelligence platforms for the prediction of the probability of malignant transformation of oral potentially malignant disorders are required as adjunctive decision-making platforms in contemporary clinical practice. This study utilized time-to-event learning models to predict malignant transformation in oral leukoplakia and oral lichenoid lesions. A total of 1098 patients with oral white lesions from two institutions were included in this study. In all, 26 features available from electronic health records were used to train four learning algorithms—Cox-Time, DeepHit, DeepSurv, random survival forest (RSF)—and one standard statistical method—Cox proportional hazards model. Discriminatory performance, calibration of survival estimates, and model stability were assessed using a concordance index (c-index), integrated Brier score (IBS), and standard deviation of the averaged c-index and IBS following training cross-validation. This study found that DeepSurv (c-index: 0.95, IBS: 0.04) and RSF (c-index: 0.91, IBS: 0.03) were the two outperforming models based on discrimination and calibration following internal validation. However, DeepSurv was more stable than RSF upon cross-validation. External validation confirmed the utility of DeepSurv for discrimination (c-index—0.82 vs. 0.73) and RSF for individual survival estimates (0.18 vs. 0.03). We deployed the DeepSurv model to encourage incipient application in clinical practice. Overall, time-to-event models are successful in predicting the malignant transformation of oral leukoplakia and oral lichenoid lesions.

## 1. Introduction

Oral cavity cancer is the 18th most common malignancy worldwide and accounts for many head and neck cancers in contemporary clinical practice [1]. Early detection of malignancy is an important factor influencing disease morbidity and mortality following intervention [2,3]. Oral carcinogenesis may be associated with a lengthy pre-pathologic phase (between initial risk-factor exposure and overt disease onset), which features the occurrence of diseases with increased risk of malignancy, known as oral potentially malignant disorders (OPMDs). These include discreet, lesions such as leukoplakia (including proliferative verrucous leukoplakia), erythroplakia, erythroleukoplakia, and oral lichenoid lesions, together with more widespread conditions, such as oral submucous fibrosis, Plummer-Vinson syndrome, chronic discoid lupus erythematosus, and dyskeratosis congenita [4]. Appropriate recognition and management of OPMDs are essential to ensure early recognition of malignancy, delivery of effective treatment with reduced morbidity, and, ultimately, to improve long-term prognosis and survival for oral cancer patients.

Malignant transformation potential (MTP) of OPMDs, unfortunately, varies substantially between 0.13 and 85%, according to the clinical subtype [5,6,7,8]. For example, proliferative verrucous leukoplakia and erythroplakia, although relatively rare in clinical practice, are known to exhibit the highest MTPs [5,6,7,8], whilst other more common lesions, such as leukoplakia or oral lichenoid lesions, demonstrate highly equivocal transformation potentials. Clinico-pathological characterization of OPMDs, including the presence and extent of epithelial dysplasia, anatomical location, lesion size and appearance, together with various systemic comorbidities, have been studied, respectively, as the key features influencing malignant transformation risk [9,10,11]. To date, however, platforms that encourage accurate prediction of transformation risk for such lesions on an individual basis remain elusive.

Artificial intelligence and machine learning are now increasingly applied to the prediction of oral oncological outcomes [12]. These algorithms provide automated and exclusive prediction or classification of clinical outcomes upon learning and detecting patterns from health data without being outrightly programmed by the user to do so [13]. Many products based on this technology are being applied in precision medicine to support clinical decision making and encourage individualized treatment selection and monitoring regimens for patients [14]. In the context of oral squamous malignancies, most models have considered clinical outcomes, such as cell-type recognition, treatment response, occult metastasis, and disease prognosis, more than the malignant transformation of OMPDs [12,15]. Furthermore, the very few learning models currently proposed for OPMD malignant transformation have considered outcomes as purely binary classes (likely or unlikely), rather than dynamic variables that incorporate time-to-event data or generate outcomes as a probability of transformation over time, which would be more clinically useful [16,17]. Therefore, this study sought to compare and validate supervised deep and conventional learning algorithms for the risk-probability prediction of malignant transformation in OPMDs. The rationale for this comparative approach was to determine the utility of the deep learning approaches against conventional tree-based and statistical methods in other to select the optimal model for further validation and preliminary deployment in practice. We hypothesized that the deep-learning methods will have balanced performance accuracy and stability compared to conventional machine-learning or statistical models.

## 2. Materials and Methods

### 2.1. Patients and Dataset

Data from 716 patients with a clinical diagnosis of oral leukoplakia, oral lichen planus, or oral lichenoid lesions who underwent incisional or excisional biopsy between 1 January 2003, and 31 December 2019 were obtained from the Hong Kong Hospital Authority Clinical Management System (HA-CMS) of the Queen Mary Hospital, Hong Kong. These patients were encountered across the Head and Neck Surgery, Otorhinolaryngology, and Oral and Maxillofacial Surgery services of the institution. Included patients were those with a minimum follow-up of 18 months. However, patients with synchronous erythroplakia and proliferative verrucous leukoplakia or those with previous oral cavity cancers before the data-collection time frame were excluded from the study. Demographic, clinical, pathologic, and treatment information of suitable patients was collected from the HA-CMS electronic health record. The specific features retrieved are listed in Table 1. These features have been presented in several reports as independent risk predictors for malignant transformation of these oral leukoplakia and oral lichenoid lesions [9,10,18,19]. Key dates included the date of histologic diagnosis and the date of malignant transformation, if any. The censoring date used was 15 August 2021. The outcome considered in this study was the time to malignant transformation of oral leukoplakia and oral lichenoid lesions. Hence, the output of the models is interpreted as the probability of being free of malignant transformation at each time point or period from the date of histologic diagnosis. Only oral squamous cell carcinomas arising within the lesion focus were considered relevant malignancies in this study. Moreover, tumors identified as microinvasive or superficially invasive on histology were included, while carcinoma in situ was considered a severe type of epithelial dysplasia without stromal invasion, in line with the most recent WHO criteria for grading of oral epithelial dysplasia [20].

### 2.2. Data Cleaning and Feature Engineering

Electronic spreadsheets were used for data entry, with each column filtered to ensure correlation of variables and identification of missing instances. Input variables were either continuous, ordinal, nominal, or binary (Table 1). Three features (family history of malignancies, size of the lesion, and lesion border status) had between 64.8% and 94.8% of variables missing and were excluded from further analysis. One-hot transformation of the smoking and alcohol-consumption risk-habit categories was performed to engineer a new feature that differentiated patients into non-smoking, non-alcohol-drinking (NSND) patients and smoking and alcohol-drinking (SD) patients. The rationale for this stratification has already been described by our group and others [21,22]. No data transformation or feature engineering was performed with other categorical input features. Neither standardization nor normalization was performed for the age of patients at diagnosis or the Charlson comorbidity index, which represented the continuous features for modeling, as they did not improve the performance metrics during experimentation.

### 2.3. Machine Learning Algorithms

Five algorithms, including two standard classifiers and three neural-network-extended models were compared to determine their suitability to model the probability of malignant transformation over time. Detailed description of the architecture of each algorithm has been described in our previous report [23]. DeepSurv, time-dependent neural net cox model (Cox-Time), and DeepHit were the configurable deep-learning models used for training, while random survival forest (RSF) and the Cox proportional hazard (Cox-PH) model were used for comparison, as the performance of the latter methods had not been previously considered for malignant-transformation prediction. DeepSurv is a non-linear, feed-forward neural-network-based extension of the standard Cox regression model that fulfils the proportional-hazards assumption, while Cox-Time represents the nonproportional neural-net transformation of the Cox model with time-varying input variables [24]. While both DeepSurv and Cox-Time are continuous-time algorithms, DeepHit was implemented to serve as the non-proportional discrete-time extension of these models [25,26]. This was to explore whether the continuous-time models were restrictive in determining the discriminatory performance and calibration of risk probabilities obtained for this outcome. RSF, which represents a robust learning method that grows the trees by variable subset selection at each node, was the comparative ensemble learning model against which the performance of the deep-learning models were further compared [27].

### 2.4. Model Training and Internal Validation

Data were split into train and validation sets based on the 80:20 rule. Training data were resampled using five-fold cross-validation, with performance estimates generated for each stratum. Hyperparameters for the neural networks, i.e., learning rate, number of hidden layers, nodes per layer, drop-out, and batch size, were tuned based on the performance measures at the algorithm level. The different hyperparameters considered are presented in Table S1. Additionally, early stopping regularization was implemented in the deep-learning models to deter model training when there was no improvement on the validation fold. Mean and standard deviations of the performance measures obtained across the five cross-validation folds were used to assess and compare the stability of the algorithm on different datasets. The internal validation cohort unseen during training and cross-validation was selected randomly using computer-generated serial numbers. Performance measures generated from the internal-validation dataset were the basis for comparison of the algorithms in this study.

### 2.5. Model Performance Measures

Both the discriminative performance and calibration of the models for malignant-transformation forecasting were assessed. Harrell’s concordance index (c-index) was used as the measure of model discrimination when the order-of-probability estimates per follow-up time were considered for random pairs. Scores range from 0 to 1, with a value of 0.5 representing random discrimination. In addition to the c-index, the integrated Brier score (IBS) considering all represented time points in the training data was used to compare the accuracy of the predicted probabilities among algorithms. A lower IBS denotes better calibration, and only models with scores below 0.25 are deemed useful in real-world scenarios [25].

### 2.6. External Validation and Algorithm Deployment

To validate the best-performing model(s), this study utilized a previously published dataset of 590 patients with OPMDs treated by laser surgery at the Maxillofacial Surgery Unit of the Newcastle Dental Hospital and the Royal Victoria Infirmary between August 1996 and December 2014 [8,28,29]. Patients with erythroplakia and proliferative verrucous leukoplakia were excluded. Further, those with an unexpected diagnosis of squamous malignancy following a preliminary diagnosis of dysplasia from incisional biopsy were not included in the external validation cohort. In total, 382 patients were used for analysis (Table S2). As there were missing features in these data compared to the original training and internal validation, we examined the effect of this scenario on the performance of the outperforming prediction models by re-training and re-validating the models on these features before external validation. In line with the recent proposition for real world application of promising machine-learning models [30], we performed a web-based deployment of the best-performing algorithm considering the discrimination, calibration, and stability measures obtained during both validation procedures.

### 2.7. Computation

Descriptive statistics were performed using SPSS v 26 (IBM, Armonk, NY, USA). Training, testing, and validation of the deep, ensemble, and standard Cox models, as well as interactive graphic user interface for day-to-day application and further validation in clinical oncological centers and general practices, were performed with Python v 3.8.7 (Python Software Foundation, Wilmington, DE, USA) [24,25,27].

## 3. Results

### 3.1. Patient Characteristics

Seven hundred and sixteen patients with oral leukoplakia and lichenoid lesions were utilized for model training and internal validation. Descriptive data of this cohort are presented in Table 2. Patients were between 18 and 89 years of age, with more females (56.0%) than males (44.0%). A majority of the patients were NSND (65.5%), and only a few of the SD patients (2.0%) who indulged in the risk habits at diagnosis continued with their use afterward. The mean Charlson comorbidity index of this cohort was 0.64, with a higher prevalence of hypertension (29.5%) than hyperlipidemia (17.0%), diabetes mellitus (15.5%), and autoimmune diseases (5.9%).

More oral leukoplakia cases than oral lichenoid lesion cases were included (54.3% vs. 45.7%). Of those with oral lichenoid lesions, the erosive clinical subtype was mostly represented (19.8%) than the asymptomatic reticular or papular variants (14.0%), as this often warranted an incisional or excision biopsy at our institution. Most lesions involved the buccal or labial mucosa (56.8%) and were solitary (65.5%). Four hundred and sixteen patients received treatment that was either surgical (30.9%) or pharmacological (27.2%), and 19% of the patients treated via surgical excision experienced between one and four recurrences. Epithelial dysplasia was present in 9.5% and 7.0% of the lesions at diagnosis and during follow-up biopsies, respectively. Overall, 10.6% of the patients developed oral squamous cell carcinoma emanating from the lesions, with an average follow-up time of 90.9 months. A majority of the cancers were early-stage tumors (7.9%), and most patients (8.9%) were in remission as of the censoring date.

### 3.2. Performance of Time-to-Event Machine-Learning Models

Following data splitting, 573 patients were used for training and five-fold cross-validation of the algorithms, while internal validation was performed using 143 randomly selected patients who were unseen during model training. The metrics of each algorithm on these datasets are shown below.

#### 3.2.1. Cox-PH

**Figure 1:**
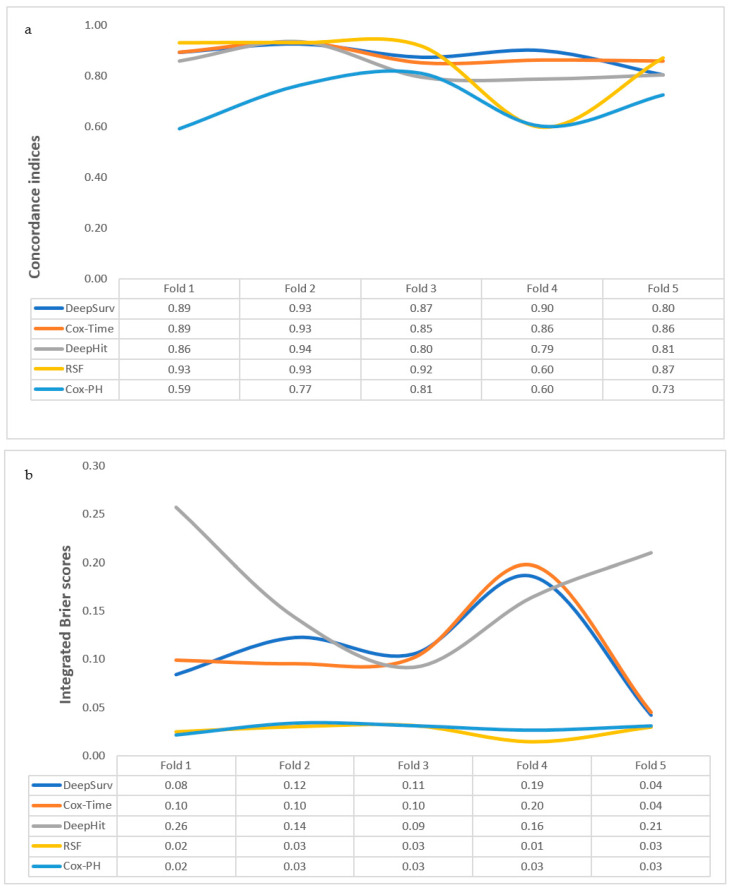
(**a**) Concordance indices across the five cross-validation folds for algorithms trained for prediction of malignant transformation. (**b**) Integrated Brier scores across the five cross-validation folds for algorithms trained for prediction of malignant transformation.

Compared to the IBS, concordance indices across the cross-validation fold were less stable with this model (Figure 1). The average c-index and IBS following cross-validation were 0.70 and 0.03, respectively, while performance metrics on the unseen data obtained were a c-index of 0.83 and an IBS of 0.03, respectively.

#### 3.2.2. Cox-Time

**Figure 2:**
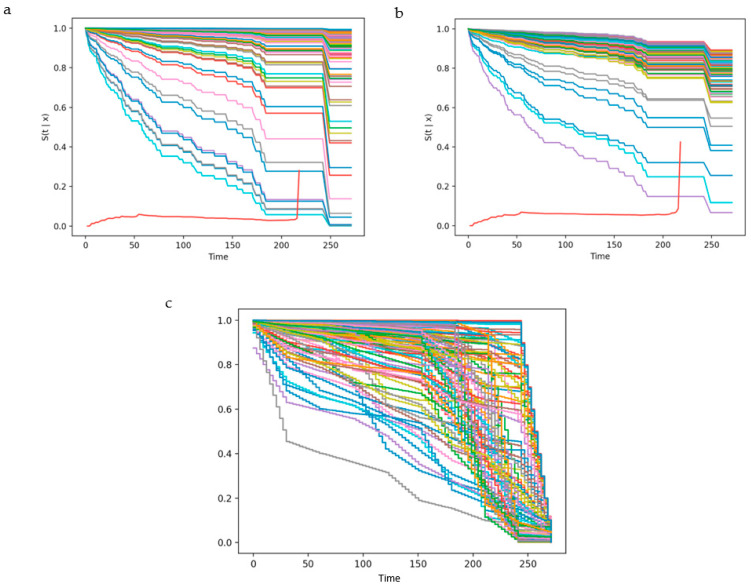
Predicted malignant-transformation-free survival plots generated for 143 patients in the internal validation cohort for (**a**) DeepSurv, (**b**) Cox-Time, and (**c**) DeepHit. DeepHit plots were generated following linear interpolation. The red lines in (**a**,**b**) represent the Brier scores plotted at each time point.

The discriminative performance of Cox-Time was stable, while the IBS scores across five folds were fairly unstable (Figure 1). The mean c-index and IBS following cross-validation were 0.88 and 0.11, respectively. Additionally, the model performance measures on internal validation were 0.86 for c-index and 0.06 for IBS (Table 3). The predicted probability function for each patient in the validation cohort is plotted in Figure 2.

#### 3.2.3. DeepHit

Concordance indices were relatively more stable than IBS scores across the training data folds for this model (Figure 1). C-index and IBS following cross-validation were 0.84 and 0.17, respectively, while on internal validation, scores of 0.86 for c-index and 0.08 were obtained, respectively (Table 3). Predicted probability functions for patients in the validation cohort are plotted in Figure 2.

#### 3.2.4. DeepSurv

Integrated Brier scores were less stable compared to c-indices for the cross-validation folds (Figure 1). The mean c-index and IBS were 0.88 and 0.11, respectively (Table 3). Upon internal validation, better c-index and integrated Brier scores of 0.95 and 0.04, respectively, were obtained. Estimated probability functions for patients in the validation cohort are plotted in Figure 2.

#### 3.2.5. RSF

Concordance indices were less stable than integrated Brier scores across the training data folds (Figure 1). C-index and IBS values were 0.85 and 0.03 for cross-validation and 0.91 and 0.03 following internal validation, respectively (Table 3).

### 3.3. Comparing the Performance Measures of the Algorithms

Regarding the stability of the algorithms in handling different datasets, we observed that Cox-Time and DeepSurv were the most stable algorithms for assessment of discriminative tasks, while the standard Cox-PH was stable for obtaining calibrated probability estimates over time. RSF was the least stable algorithm for discriminative tasks, while DeepHit was the least stable based on the integrated Brier scores. Overall, DeepSurv had the best concordance index, while RSF had the lowest integrated Brier scores, as assessed on the internal validation cohort. However, the IBS of RSF was only slightly better than Cox-PH and DeepSurv (Table 3). DeepHit also had the worst integrated Brier score, although this is still very acceptable in practice (i.e., <0.25), while the standard Cox-PH model had the poorest performance based on model discrimination.

### 3.4. External Validation and Effect of Missing Variables on Trained Models

The two best-performing algorithms for model discrimination and calibration, i.e., DeepSurv and RSF, were subjected to external validation using the Newcastle OPMD cohort. Prior to that, both models were re-trained to assess the effect of the missing variables on the model performance. Both the discrimination and calibration of DeepSurv were affected following re-training, with lower mean c-index and IBS scores upon cross-validation (Table 3). However, the reverse was the case for RSF, which obtained slightly better estimates than the full model. Upon internal validation, the performance of both models was similar, albeit slightly lower than the metrics obtained with the original models. External validation of the re-trained models obtained respective c-index and IBS scores of 0.82 and 0.18 for DeepSurv, while for RSF, performance scores were 0.73 for discrimination and 0.03 for calibration.

### 3.5. Algorithm Deployment

**Figure 3:**
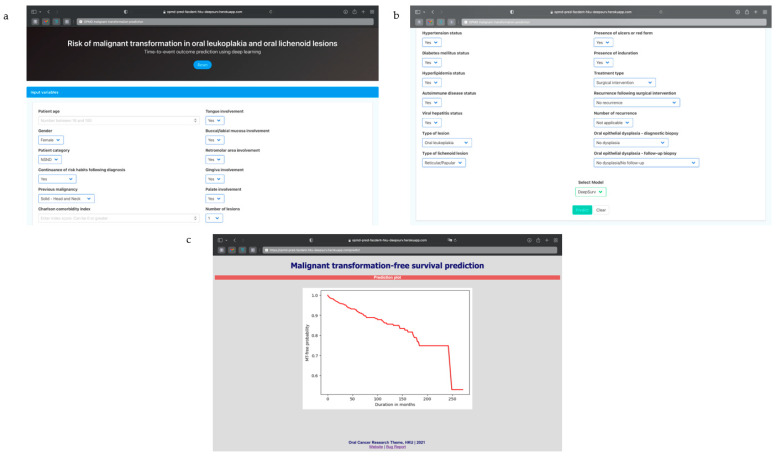
Preview of web-based prognostic tool generated from the model for optimization. (**a**,**b**) HTML page for input of predictive variables; (**c**) display of output generated upon prediction.

We deployed the DeepSurv algorithm using the Flask module in Python to create an interactive web-based tool for practical use, similar to tools developed by other authors [31]. Visuals on the functionality and output of the application are presented in Figure 3. The application, which is primarily for research or informational purposes, can be assessed publicly at https://opmd-pred-facdent-hku-deepsurv.herokuapp.com (accessed on 5 November 2021). Codes used for this production can also be found at https://github.com/jaadeoye/opmd-mt-deepsurv-app (accessed on 5 November 2021) for potential modification in respective institutions.

## 4. Discussion

Prediction of the malignant transformation of OPMDs is critical to the prevention and early diagnosis of oral squamous cell carcinoma. Currently, there are no concrete decision-making support platforms to assist clinicians in the management of OPMDs [15]. Due to the highly variable malignant-transformation potentials reported for oral leukoplakia and oral lichenoid lesions, an effective platform would help clinicians rationalize the choice of treatment intervention and deliver appropriate patient follow-up and long-term monitoring arrangements [15]. As artificial intelligence is increasingly being applied to oncological decision making and outcome prediction, this study presents the comparison and validation of deep and tree-based time-to-event machine-learning algorithms to predict malignant-transformation-free survival of patients with oral leukoplakia and oral lichenoid lesions.

This study found DeepSurv and RSF to be robust for discrimination and provision of better-calibrated probability estimates as a function of time for the malignant transformation of oral leukoplakia and lichenoid lesions. This means that clinical scenarios involving the comparison of malignant-transformation probability estimates among patients for treatment selection, risk stratification, and disease surveillance plans are better performed using DeepSurv, while individual survival distributions are only slightly better modeled relative to the actual probability functions using the RSF model. The former may be attributed to the implementation of DeepSurv specifically to predict individuals’ risk before treatment recommendation, thus prioritizing discriminative performance over calibrated probability estimates [24,25]. Furthermore, this finding is in keeping with a previous implementation of these algorithms to train prognostic features to predict the prognoses of oral squamous malignancies [23,32]. Nonetheless, DeepSurv had very satisfactory calibration estimates, which can permit its singular use for both tasks in practice. While our findings support the use of both DeepSurv and RSF based on the clinical tasks to be performed, our analysis showed that the RSF model is still very unstable for discriminatory tasks, which may mean equivocal performance with changes in the modeling dataset. However, this was notable with the use of an expanded than reduced number of features implemented during model re-training.

Upon comparing the best-performing machine-learning models with clinical nomograms for prediction of malignant transformation of OPMDs, DeepSurv outperformed both existing nomograms, especially with regards to discrimination [18,33]. External validation of the best-performing algorithms in this study suggests that these models are reliable, with reproducible performances in other populations with disparate sociodemographic characteristics and risk profiles. However, we found that the ordering of the risk probabilities and the accuracy of predicted survival functions were affected differently upon re-training due to the missing features. While external validation was satisfactory for DeepSurv, the calibration estimate was higher that than obtained in internal validation, although within satisfactory limits. Likewise, poorer discrimination was observed for RSF while retaining its ability to provide near-actual estimates. With this observation, we propose that better estimates can be obtained if all variables used for model training are included. Additionally, these studies may consider incorporating techniques for handling missing features and instances specific to supervised learning for discriminative tasks involving the models [34,35].

Though this study pioneered machine-learning models for prediction of malignant transformation of oral leukoplakia and oral lichenoid lesions, it is not without limitations. First, three input variables were excluded from model training, which, if included, may have further improved the predictive performance and stability of the models. However, the current performance estimates are satisfactory, pending further validatory endeavors and clinical deployment. Second, the retrospective design of this study and lack of direct patient recruitment may hamper the reliability of the input features used. However, instances obtained for each patient were verified across several clinical specialty platforms to ensure their accuracy prior to data entry. Third, the prediction time points and frame of the models were restricted to the duration of patients’ follow-up in the training models, with forecasts only available until 271 months following histologic diagnosis. Even so, poorer calibration may be experienced with the use of the interactive web-based tool at time points above 210 months due to a reduced number of patients with longer follow-up. Last, the study did not consider molecular data, which may improve the clinical performance of the machine-learning models [15]. Future studies should consider prospective validation of these models while including results from biomarker assays to deliver enhanced and more precise predictive ability.

## 5. Conclusions

This study successfully utilized time-to-event algorithms to model the malignant-transformation risk for oral leukoplakia and oral lichenoid lesions. The DeepSurv algorithm had the best discriminative performance, while RSF outperformed other models, with better-calibrated probability estimates. External validation of both models was satisfactory, which shows promise for application in contemporary oncology, as well as general medical and dental practices, especially in areas where access to specialist clinical expertise may be lacking.

## Figures and Tables

**Table 1:** Input features, variable category, and missing data.

Input Feature	Type	Missing Instance	Handling Technique
Age	Continuous	0	NA
Sex	Binary	0	NA
Tobacco smoking	Binary	2	One-hot transformation
Alcohol drinking	Categorical (nominal)	33	
Patient category	Categorical (nominal)	0	NA
Risk-habit indulgence following diagnosis	Categorical (nominal)	0	NA
Previous malignancy	Categorical (nominal)	0	NA
Charlson Comorbidity Index (CCI)	Continuous	0	NA
Hypertension status	Binary	0	NA
Diabetes Mellitus status	Binary	0	NA
Hyperlipidemia status	Binary	0	NA
Autoimmune disease status	Binary	0	NA
Viral hepatitis status	Binary	0	NA
Family history of malignancy	Binary	592	Variable elimination
Type of lesion	Binary	0	NA
Clinical subtype of lichenoid lesion	Categorical (nominal)	0	NA
Tongue/FOM involved	Binary	0	NA
Labial/buccal mucosa involved	Binary	0	NA
Retromolar area involved	Binary	0	NA
Gingiva involved	Binary	0	NA
Palate involved	Binary	0	NA
Number of lesions	Categorical (ordinal)	0	NA
Lesion size	Continuous	464	Variable elimination
Presence of ulcers or erosions	Binary	0	NA
Lesion border status	Binary	679	Variable elimination
Presence of induration	Binary	0	NA
Treatment at diagnosis	Categorical (nominal)	0	NA
Recurrence after surgical excision	Binary	0	NA
Number of recurrences	Categorical (ordinal)	0	NA
Oral epithelial dysplasia at diagnosis	Categorical (nominal)	0	NA
Oral epithelial dysplasia detected during follow-up	Categorical (nominal)	0	NA

NA—Not applicable; FOM—Floor of the mouth.

**Table 2:** Demographic, clinical, and pathologic characteristics of all patients with oral leukoplakia and lichenoid lesions used to train learning algorithms.

Variables		N = 716
		N (%)
Median age (IQR)		58 (49–67)
Gender	Female	401 (56.0)
	Male	315 (44.0)
Patient category	NSND	469 (65.5)
	SD	247 (34.5)
Continued risk habits following diagnosis	Yes	14 (2.0)
	No	167 (23.3)
	Not applicable	535 (74.7)
Previous malignancy	Head and neck tumors	21 (2.9)
	Other tumors	46 (6.4)
	Hematologic malignancies	23 (3.2)
	No malignancy	626 (87.4)
Charlson comorbidity index—mean (SD)		0.64 (1.02)
Hypertension		211 (29.5)
Diabetes mellitus		111 (15.5)
Hyperlipidemia		122 (17.0)
Autoimmune disease		42 (5.9)
Viral hepatitis infection		69 (9.6)
Lesion	Oral leukoplakia	389 (54.3)
	Oral lichen planus/oral lichenoid lesion	327 (45.7)
Clinical subtype of lichenoid lesion	Reticular/Papular	100 (14.0)
	Erosive/Atrophic	142 (19.8)
	Plaque	85 (11.9)
Tongue/FOM		245 (34.2)
Buccal/Labial mucosa		407 (56.8)
Retromolar area		26 (3.6)
Gingiva		88 (12.3)
Palate		23 (3.2)
Number of lesions	Single	469 (65.5)
	Bilateral or double	210 (29.3)
	Multiple	37 (5.2)
Presence of ulcers or erosions		228 (31.8)
Induration		47 (6.6)
Treatment	Surgical excision	221 (30.9)
	Medical	195 (27.2)
	No treatment	300 (41.9)
Post-excision recurrence		42 (19.0)
Number of recurrences	1	30 (4.2)
	2	7 (1.0)
	3	4 (0.6)
	4	1 (0.1)
Oral epithelial dysplasia at diagnosis	Absent	641 (89.5)
	Mild	34 (4.7)
	Moderate	27 (3.8)
	Severe	7 (1.0)
	Unknown (defaulted biopsy at diagnosis)	7 (1.0)
Oral epithelial dysplasia at follow-up	Absent	658 (91.9)
	Mild	11 (1.5)
	Moderate	15 (2.1)
	Severe	24 (3.4)
	Unknown (defaulted biopsy during follow-up)	8 (1.1)
Malignant transformation		76 (10.6)
AJCC TNM stage	Stage I	47 (6.6)
	Stage II	9 (1.3)
	Stage III	6 (0.8)
	Stage IV	12 (1.7)
Tumor grade	Well differentiated	23 (3.2)
	Moderately differentiated	30 (4.2)
	Poorly differentiated	3 (0.4)
Tumor prognosis	Remission	58 (8.1)
	Recurrence	6 (0.8)
	Cancer-related death	6 (0.8)
	Second primary tumor	6 (0.8)

**Table 3:** Performance measures of time-to-event algorithms for prediction of malignant transformation of oral leukoplakia and lichenoid lesions.

Models	Five-Fold Cross-Validation		Internal Validation		Repeat Five-Fold Cross-Validation with Reduced Features		Internal Validation		External Validation	
	Concordance Index	Integrated Brier Scores (IBS)	Concordance Index	Integrated Brier Scores (IBS)	Concordance Index	Integrated Brier Scores (IBS)	Concordance Index	Integrated Brier Scores (IBS)	Concordance Index	Integrated Brier Scores (IBS)
	Mean (SD)	Mean (SD)			Mean (SD)	Mean (SD)				
Cox-PH	0.70 (0.098)	0.03 (0.005)	0.83	0.03						
Cox-Time	0.88 (0.034)	0.11 (0.055)	0.86	0.06						
DeepHit	0.84 (0.061)	0.17 (0.064)	0.86	0.08						
DeepSurv	0.88 (0.046)	0.11 (0.053)	0.95	0.04	0.78 (0.097)	0.13 (0.069)	0.92	0.05	0.82	0.18
RSF	0.85 (0.142)	0.03 (0.007)	0.91	0.03	0.89 (0.064)	0.03 (0.006)	0.92	0.03	0.73	0.03

## Data Availability

The datasets generated and/or analyzed during the current study are not publicly available due to the need to maintain patient confidentiality as some of the patients are still in review. However, they may be made available by the corresponding authors on reasonable request.

## Supplementary Materials

The following are available online at https://www.mdpi.com/article/10.3390/cancers13236054/s1. Table S1: Tuning hyperparameters for the deep learning algorithms; Table S2: Demographic, clinical, and pathologic characteristics of the external validation cohort.
