# Supplementary material for: Deep Learning Predicts the Malignant-Transformation-Free Survival of Oral Potentially Malignant Disorders
Source: Cancers (Basel). 2021 Dec 1;13(23):6054. doi: 10.3390/cancers13236054 (PMC8657223; doi:10.3390/cancers13236054)
Supplement: Supplementary file 1 [file cancers-13-06054-s001.zip › cancers-1474072-supplementary.pdf]

cancers

MDPI

Article

Deep Learning Predicts the Malignant-Transformation-Free Survival of Oral Potentially Malignant Disorders

John Adeoye [1], Mohamad Koohi-Moghadam [2], Anthony Wing Ip Lo [3], Raymond King-Yin Tsang [4], Velda Ling Yu Chow [5], Li-Wu Zheng [1], Siu-Wai Choi [1], Peter Thomson [6,*] and Yu-Xiong Su [1,*]

Table S1. Tuning hyperparameters for the deep learning algorithms.

Hyperparameters	Range
Optimizer	ADAM, SGD
Activation	Rectifier linear unit (ReLU)
Hidden layers	1–4
Nodes per layer	32, 64, 128, 256
Dropout	0.1–0.3
Batch size	64, 128, 256
Learning rate (LR)	Hyperparameter search and optimal LR used within the model
Alpha (DeepHit)	0.2
Sigma (DeepHit)	0.1

ADAM – Adaptive moment estimation; SGD – Stochastic gradient descent.

Table S2. Demographic, clinical, and pathologic characteristics of the external validation cohort.

**Variables**		**N = 382**
		**N (%)**
**Median age (IQR)**		58 (50–69)
Gender	Female	166 (43.5)
	Male	216 (56.5)
Patient category	NSND	Missing
	SD	
Continued risk habits following diagnosis	Yes	Missing
	No	
	Not applicable	
Previous malignancy	Head and neck tumors	Missing
	Other tumors	
	Hematologic malignancies	
	No malignancy	
Charlson comorbidity index – mean (SD)		Missing
Hypertension		Missing
Diabetes mellitus		Missing
Hyperlipidemia		Missing
Autoimmune disease		Missing
Viral hepatitis infection		Missing
Lesion	Oral leukoplakia	292 (76.4)
	Oral lichenoid lesion	90 (23.6)
Clinical subtype of lichenoid lesion	Reticular / Papular	26 (6.8)
	Erosive/Atrophic	15 (3.9)
	Plaque	49 (12.8)
Tongue / FOM		263(68.8)
Buccal / Labial mucosa		58 (15.2)
Retromolar area		3 (0.8)
Gingiva		29 (7.6)
Palate		45(11.8)
Number of lesions	Single	Missing
	Bilateral or double	
	Multiple	
Presence of ulcers or erosions		61 (16.0)
Induration		Missing
Treatment	Surgical excision	382 (100.0)
Post-excision recurrence		52 (13.6)
Number of recurrences	1	34 (8.9)
	2	11(2.9)
	3	4 (1.0)
	4	2 (0.5)
	7	1 (0.3)
Oral epithelial dysplasia at diagnosis	Absent	42 (11.0)
	Mild	113 (29.6)
	Moderate	114 (29.8)
	Severe	113 (29.6)
Oral epithelial dysplasia at follow-up	Absent	335 (87.7)
	Mild	11(2.9)

	Moderate	11 (2.9)
	Severe	17 (4.5)
	Unknown	8 (2.1)
Malignant transformation		22 (5.8)

IQR – Interquartile range; NSND – nonsmoker non-drinker; SD – smoker and/or drinker; SD – standard deviation; FOM – floor of the mouth; AJCC – American Joint Committee on Cancer.
